# Malignant head and neck paragangliomas: Is there an optimal treatment strategy?

**DOI:** 10.1186/1758-3284-2-23

**Published:** 2010-09-23

**Authors:** Daniel J Moskovic, Joseph R Smolarz, Douglas Stanley, Camilo Jimenez, Michelle D Williams, Ehab Y Hanna, Michael E Kupferman

**Affiliations:** 1 Scott Department of Urology, Baylor College of Medicine, Houston, Texas, USA; 2 Columbia Business School, New York, New York, USA; 3 Department of Otorhinolaryngology - Head and Neck Surgery/Otolaryngology, The University of Texas Health Science Center at Houston, Houston, Texas, USA; 4 Department of Endocrine Neoplasia and HD, The University of Texas, M. D. Anderson Cancer Center, Houston, Texas, USA; 5 Department of Pathology, The University of Texas, M. D. Anderson Cancer Center, Houston, Texas, USA; 6 Department of Head and Neck Surgery, The University of Texas, M. D. Anderson Cancer Center, Houston, Texas, USA

## Abstract

**Background:**

Little is known about management and prognosis for malignant head & neck paragangliomas. We reviewed records of these patients to determine optimal treatment strategies.

**Methods:**

We reviewed 113 cases of head & neck paragangliomas treated at our institution from 1970 to 2005. Nineteen patients were included in the study. All had primary surgical treatment at another institution. Metastatic disease was treated with radiation, chemotherapy, or both. Survival and complications were evaluated. P values were determined by Fischer's exact test.

**Results:**

All patients treated with chemotherapy and radiation age ≥ 40 years had disease progression. Of the patients < 40, two had stable disease; one had regression of disease with treatment. Patients without disease progression had better prognosis and were alive at last follow-up.

**Conclusions:**

Clinical benefit was derived from aggressive treatment. However, careful consideration of the risks of observation versus intensive therapy should be undertaken when managing these patients.

## Background

Paragangliomas (PGs) represent rare tumors of neuroendocrine origin, are comprised of sustenacular and chief cells in an organoid or Zellballen pattern and commonly arise in various locations in the head and neck [1]. Malignant PGs are defined by presence of pathologically confirmed PG cells in lymph nodes or in distant organs, rather than by the histological features of the primary tumor [2]. Only a small percentage of patients ultimately develop metastatic disease, and malignant PGs are believed to represent only 4% to 15% of all head and neck PGs [3-5]. When considering the major classes of PG sub-types, rates of malignancy are believed to be 2 - 4% for jugular-tympanic tumors, 6% for carotid body tumors, and 16 - 19% for vagal tumors [6,7].

For patients identified with regionally metastatic disease, radiation after primary surgical treatment offers the best prognosis [4]. Patients with metastases to distant organs, most commonly lung and liver, are often treated with systemic therapy [8]. However, due to the rarity of these lesions, optimal treatment strategies have not been defined to date. In particular, most studies of chemotherapy for malignant PG have focused on thoracic and abdominal PG, which have biological behaviors distinct from head and neck PG [9]. Abdominal, pelvic, and thoracic PGs originate in the sympathetic autonomic nervous system ganglia. They are frequently associated with a norepinephrine biochemical phenotype. A significant number of sympathetic PGs are malignant. Conversely, head and neck PGs originate in parasympathetic autonomic nervous system ganglia. Typically, they are hormonally silent and are rarely malignant.

To define the outcomes and optimal treatment strategies of malignant head and neck PG, we reviewed a single institution's 35-year experience with these lesions.

## Methods

All patients with malignant PG of the head and neck treated at the University of Texas MD Anderson Cancer Center (MDACC) from 1970 to 2005 were reviewed after IRB approval was obtained. Pathology, radiologic imaging, operative reports, and all clinical notes were reviewed. Patients referred to our institution without complete documentation were excluded from our study. A comprehensive review of the literature was also performed.

## Results

### Epidemiologic Data

We identified 113 patients with cervical PGs using institutional diagnosis codes, 23 (18%) of whom had regional or distant metastases. Summary statistics are presented in Table 1. Four patients were excluded because outside hospital records were incomplete or unavailable. Thus, the records of 19 patients were available for this analysis.

**Table 1:** Summary of gender, histology, and tumor characteristics for patients in this series.

Summary Statistics	
	**(n = 19)**

***Age (mean)***	35
	
***Gender***	
Men	13 69%)
Women	6 (32%)
	
***Primary Site***	
Carotid Body	10 (53%)
Jugulotympanic	6 ( 32%)
Other	3 (16%)
	
***Metastatic Sites***	
Lymph Nodes	5 (26%)
Liver	5 (26%)
Lung	7 (37%)
Bone	13 (68%)
Other	12 (63%)

**Figure 1:**
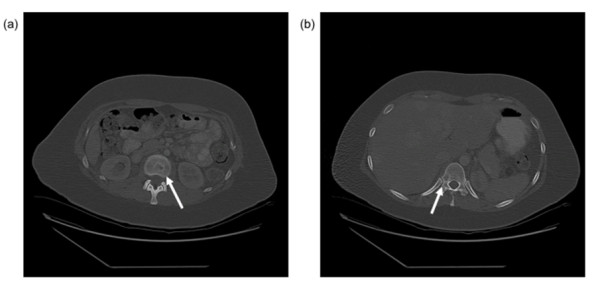
**Representative magnetic resonance image revealing bone metastasis indicated by the white arrow**.

**Figure 2:**
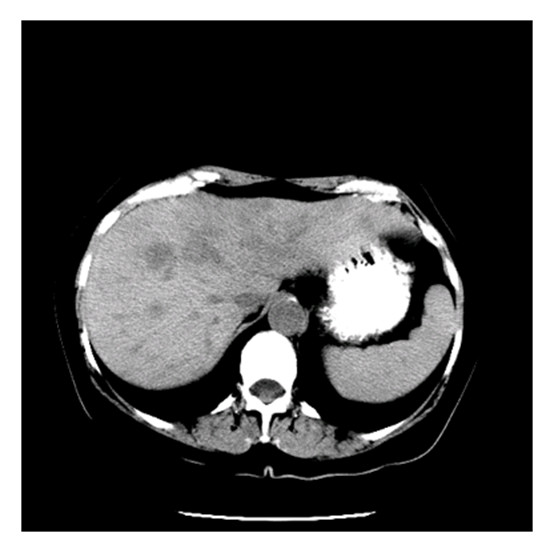
**Representative computed tomographic image revealing liver metastases in a PG patient**.

The mean age of the patients within our cohort was 35 (range: 16 - 62), with 10 (53%) patients above 40 years of age. All patients in our study had their primary surgery at outside hospitals and were referred to MDACC for recurrent or progressive disease. Our sample consisted of 10 patients (53%) with carotid body tumors, 6 (32%) with jugulotympanic tumors and 3 with other tumors (Table 1). Of note, no patients within our series presented with metastatic disease due to a primary vagal nerve PG. Bone metastases occurred most commonly in the setting of jugulotympanic tumors, whereas carotid body tumors had a predilection for both lung and bone metastases (Table 2). Figures 1 and 2 are representative radiographic images obtained from patients in this series.

**Table 2:** Summary of the extent of disease identified over the course of follow-up for patients in this series.

Tumor characteristics for patients included in this study								
		**Metastatic Sites**						
		
**Patient**	**Primary Site**	***Bone***	***Neck/Regional Nodes***	***Liver***	***Lung***	***Brain***	***Spleen***	***Eye***

1	Carotid body	+	+					
2	Middle fossa/Cavernous sinus	+						
3	Jugulotympanic		+	+	+			
4	Jugulotympanic	+				+		
5	Carotid body		+		+			
6	Carotid body	+	+					
7	Carotid body	+						
8	Jugulotympanic	+	+	+				
9	Carotid body		+	+	+			
10	Jugulotympanic	+						
11	Carotid body	+	+	+	+			
12	Jugulotympanic	+						
13	Carotid body				+			
14	Carotid body		+	+	+			
15	Carotid body	+					+	
16	Jugulotympanic	+						
17	Carotid body	+	+					
18	Carotid body/Glomus vagale				+			
19	Parotid	+						+

Review of patient records was notable for 3 patients with a family history of PG. One patient was known to have a functional tumor that led to hypertension. This patient had elevated levels of dopamine, normetanephrine and chromogranin A. One patient was known to have von Hippel-Lindau syndrome. Two patients had bilateral carotid body disease, though contralateral disease was discovered in both patients several years after carotid body tumor resection. At the time of diagnosis, genetic testing was not broadly available for patients with PGs.

### Treatment

**Figure 3:**
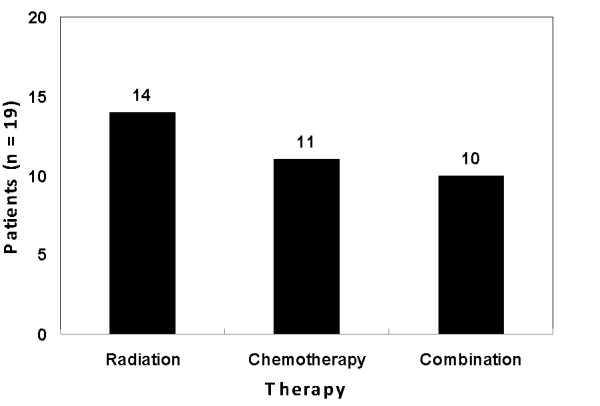
**The distribution of patients receiving radiation, chemotherapy and combination therapy**.

We examined the treatment approaches for patients within our cohort, including chemotherapy and external beam radiation therapy (EBRT) (Table 3). The most common mode of therapy was palliative radiation therapy to the spine, pelvis, and areas of local recurrence. Fourteen patients (74%) in our series received radiation and 11 patients (58%) were treated with chemotherapy. A total of 10 patients (53%) received both chemotherapy and radiation (Figure 3).

**Table 3:** Summary of individual treatments utilized over the course of follow-up at MDACC for patients in this series.

Treatments, side-effects and responses for patients included in this study								
***Gender***	***Radiation***				***Chemotherapy***			
		
	**Received**	**Dose (Gy)**	**Side Effects**	**Response**	**Received**	**Regimen**	**Side Effects**	**Response**

F	Y	143	Skin sloughing	Progression	Y	CCNU, Adriamycin	Alopecia	Progression
M	N	--	--	--	N	--	--	--
F	Y	55	Otalgia	Progression	N	--	--	--
M	Y	N/A	N/A	Progression	Y	VP-16, Cisplatin	N/A	Progression
F	Y	68	Mucositis	Progression	Y	DTIC, Adriamycin, Mesna, Ifosfamide	N/A	Progression
F	Y	N/A	N/A	Progression	Y	CDDP, VP-16	None	Progression
F	Y	N/A	N/A	Regression	Y	N/A	N/A	Regression
M	Y	90	N/A	Progression	Y	CVAD	Febrile neutropenia, N/V	Stable
F	Y	39	N/A	N/A	Y	CVAD	N/A	N/A
M	Y	60	None	Progression	Y	Adriamycin, Cytoxan, VP-16, Cisplatin, FTI	None	Progression
F	N	--	--	--	Y	CVAD	N/A	N/A
M	Y	N/A	N/A	Progression	N	--	--	--
F	N	--	--	--	N	--	--	--
F	Y	N/A	N/A	Stable	N	--	--	--
F	Y	N/A	N/A	Progression	Y	Taxol, Cytoxan, Adriamycin, DTIC	None	Stable
F	Y	N/A	N/A	Progression	N	--	--	--
F	N	--	--	--	N	--	--	--
F	N	--	--	--	N	--	--	--
F	Y	N/A	Mucositis	Progression	Y	CVAD	Alopecia	Progression

N/A implies the data are unknown, '-' implies the patients did not receive the therapy. (Summary of abbreviations: CCNU: Lomustine; VP-16: Etoposide; DTIC: Dacarbazine; CDDP: Cisplatin; CVAD: Cyclophosphamide, Vincristine, Doxorubicin, and Dacarbazine; FTI: Farnesyl transferase inhibitor; N/V: Nausea/Vomiting)

The most commonly used chemotherapy regimen was cyclophosphamide, vincristine, doxorubicin, and dacarbazine (CVAD), but this was only used in 4 patients (21%). Other chemotherapy regimens are presented in Table 3. Few treatment-related complications were reported (27%). One patient developed uncomplicated neutropenia and one patient treated with an adriamycin-based regimen developed alopecia.

Radiation doses varied widely, with patients receiving total doses of 55 Gy to 143 Gy for local control and management of metastatic sites, with 2 patients having received multiple courses of EBRT (Table 3). One patient who was treated with two courses of cervical irradiation developed skin sloughing in the setting of progressive disease. A summary of all treatments and outcomes is presented in Table 4.

**Table 4:** Characteristics, treatments and responses for patients included in this study.

Summary of tumor characteristics, treatment and responses													
	***Primary Site***			***Metastatic Sites***				***Treatment***			***Response***		
	
	**Carotid Body**	**Jugulo-tympanic**	**Other**	**Liver**	**Lung**	**Bone**	**Other**	**Radiation**	**Chemotherapy**	**Both**	**Progression**	**Stable**	**Regression**

**By Gender**													
Men	1 (17%)	4 (67%)	1 (17%)	1 (17%)	-	6 (100%)	3 (50%)	5 (83%)	4 (67%)	4 (67%)	5 (83%)	1 (17%)	-
Women	9 (69%)	2 (15%)	2 (15%)	4 (31%)	7 (54%)	7 (54%)	9 (69%)	9 (69%)	7 (54%)	6 (46%)	5 (38%)	4 (31%)	1 (8%)
													
**By Age at Presentation**													
> 40	3 (33%)	3 (33%)	3 (33%)	2 (22%)	4 (44%)	6 (67%)	6 (67%)	7 (78%)	5 (56%)	5 (56%)	7 (78%)	1 (11%)	-
< = 40	7 (70%)	3 (30%)	-	3 (33%)	3 (33%)	7 (78%)	6 (60%)	7 (70%)	6 (60%)	5 (50%)	3 (30%)	4 (40%)	1 (10%)
													
**By Primary Site**													
Carotid Body	10	-	-	3 (30%)	5 (50%)	6 (60%)	8 (80%)	-	-	-	3 (30%)	3 (30%)	1 (10%)
Jugulotympanic	-	6	-	2 (33%)	1 (17%)	5 (83%)	3 (50%)	-	-	-	5 (83%)	1 (17%)	-
Other	-	-	3	-	1 (33%)	2 (67%)	1 (33%)	-	-	-	2 (67%)	1 (33%)	-
													
**By Treatment**													
Radiation	7 (70%)	6 (100%)	1 (33%)	-	-	-	-	14	-	-	12 (92%)	1 (8%)	0 (0%)
Chemotherapy	7 (70%)	3 (50%)	1 (33%)	-	-	-	-	-	11	-	6 (55%)	2 (18%)	1 (9%)
Both	6 (60%)	3 (50%)	1 (33%)	-	-	-	-	-	-	10	6 (60%)	2 (20%)	1 (10%)

### Outcomes

**Figure 4:**
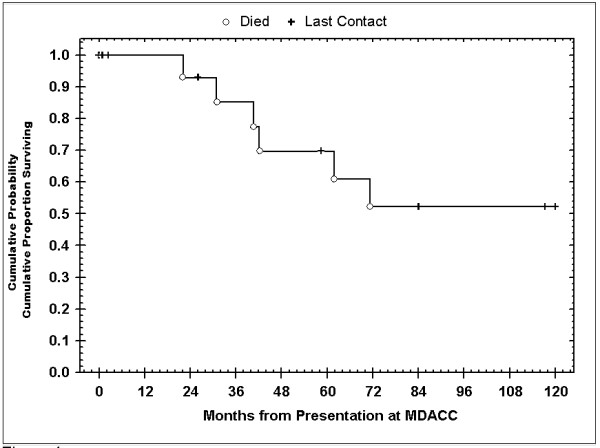
**Kaplan-Meier overall survival curves for patients based on age at diagnosis**.

Overall, five-year survival of patients above 40 years old with malignant PGs was 60% (Figure 4). Regressive and stable disease was more common in patients diagnosed before age 40, and age less than 40 years portended for improved prognosis as well. Additionally, female patients tended to have better responses to treatment compared to men (Tables 3 and 4).

Of the 14 patients treated with EBRT, 12 developed progressive disease despite therapy. One patient experienced stable disease after radiation (Tables 3 and 4). For the 11 patients (42%) treated with chemotherapy and the 10 patients receiving combination therapy, 6 (67%) had disease progression, 2 (22%) had stable disease, and 1 (11%) experienced regression. Table 4 provides a summary of outcome data stratified by treatment type.

**Figure 5:**
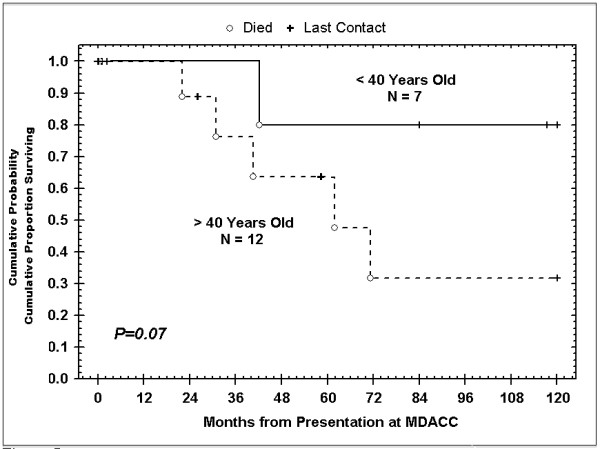
**Kaplan-Meier overall survival curves over 10 years for patients with malignant head & neck paragangliomas**.

In general, no treatment or combination of treatments demonstrated therapeutic superiority. However, patients who received radiation therapy after primary surgical treatment experienced more favorable outcomes compared to patients who did not receive radiation to control local disease (*P *= .05). During the entire follow-up period, 9 patients (47%) were found to have disease spread either to regional nodes or surrounding structures. Follow-up data revealed a 5-year survival of 84% and 10-year survival of 53% (Figure 5).

## Discussion

Due to their rarity, metastatic head and neck PGs remain clinically challenging. Our data reveal that the management and progression of malignant head and neck PGs is complicated and highly variable. These results suggest that young patients (age ≤ 40) are more likely to demonstrate a favorable response to therapy. Additionally, female patients tended to have more favorable responses, though this trend was not statistically significant. While specific regimens cannot be prescribed, the aggressive tumors may be treated with multiple systemic agents, though strict follow-up to assess progression in the absence of treatment also appears appropriate given the outcomes of treatments detailed herein.

Our data reflect the results of a previous study performed at MDACC where, in a group of 13 patients with malignant PGs (only two patients with primary head and neck PGs), 92% achieved at least stable disease on CVAD [10]. This compares to the use of a combination of cyclophosphamide, vincristine, and dacarbazine (CVD) studied in the setting of malignant PGs, where complete response was achieved in only 11% of patients, partial response in 44% of patients, and stable disease in 22% of patients [11]. A newer approach to therapy has been the use of radio-iodinated metaiodobenzylguanidine, a norepinephrine analog that has yielded a 75% 5-year survival with a majority of patients achieving at least a partial response to therapy [12-14].

Historically, external beam radiation has assumed a palliative role in the treatment of spinal and other bone metastasis [15]. None of the 14 patients receiving radiation experienced neurologic sequelae, a recognized complication in the palliative treatment of bone disease. Though not statistically significant, radiation appeared to be more effective in younger patients. These findings are in line with prior studies where radiation has been described for bony metastasis, though no evidence of a benefit to younger patients was found [16].

During the entire follow-up period, 7 patients (41%) were found to have local disease spread either to regional nodes or surrounding structures. This compares to the aforementioned study conducted by Lee et al, which revealed that 69% of patients with malignant PG experienced regional spread [4]. Though only 50% of patients with regional metastasis eventually develop distant spread of disease, these patients typically do not have favorable outcomes. High rates of local spread and poor response to therapy complicate management in these patients.

Based upon data from the National Cancer Database, Lee et al reported on patients with both regional and distant spread from head and neck PG and found a 5-year survival of 11.8% [4]. More recently, a 44% 5-year survival was observed in patients with metastatic PGs undergoing radiation and chemotherapy [12]. In a study performed by Fitzgerald et al, patients with pulmonary metastases experienced significantly shortened survival compared to patients with metastases to other sites. Our series revealed an 84% five-year survival that is likely attributable to the head and neck origin of disease in these patients compared to pheochromocytoma. Additionally, previously reported studies involve clinical trials with patients that were likely to present with more advanced disease than those reported in this series. What requires further study is the impact of germline mutations in the *SDH *genes on overall outcomes. Genotype-phenotype correlations have identified distinct mutations that pre-dispose to malignant lesions, findings that may offer novel therapies that specifically target these malignant phenotypes [17].

Though the metastatic potential of this tumor remains unclear, distant spread is an extremely rare event [18]. Therefore, regional lymphadenectomy, as well as adjuvant radiation with primary surgical resection for metastatic PG, remains the optimal treatment modality. Data from the National Cancer Database revealed that 34% of patients with regional metastasis received adjuvant radiation, though the distribution of patients receiving radiation increases with time [4]. Due to the inability to detect aggressive disease, surgical resection, radiation therapy, or observation without adjuvant therapy is currently the standard of care for the tumors that do not exhibit aggressive characteristics.

**Figure 6:**
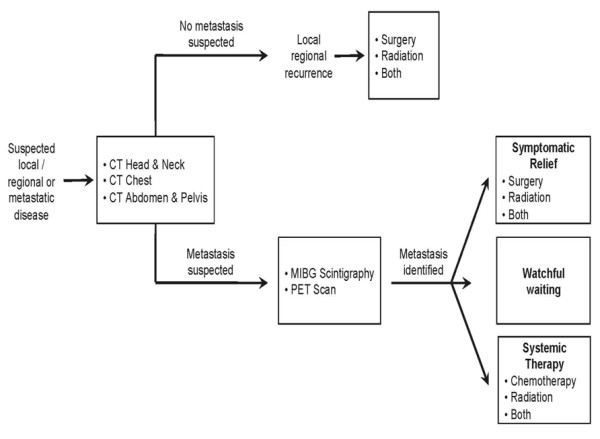
**Algorithmic overview of diagnostic and treatment options of patients with suspected local/regional and metastatic disease**. CT = computed tomography, PET = positron emission tomography, MIBG = metaiodobenzylguanidine.

The present management of malignant head and neck PGs are based largely on retrospective data. Due to the rarity of this disease process, prospective data are difficult to obtain. The 35-year interval over which these patients presented complicates the ability to compare the efficacy of any particular management approach and compromises the completeness of the patient records. Additionally, due to the small number of patients presenting with head and neck PGs, some of the trends described here were unable to achieve statistical significance. Based on our observations we have developed a treatment algorithm that guides, at a very high level, the diagnostic and treatment options for patients. However, lacking controlled trial data prevent the recommendation of specific therapeutic modalities or types of therapy within each category. Figure 6 presents this algorithmic overview.

## Conclusions

What remains clear is that aggressive treatment of regional and distant disease with radiation therapy and systemic chemotherapy may be a viable option to achieve disease control in a large number of patients. In our study, 33% of patients experienced clinical benefit with aggressive therapy. Therefore, the risks and benefits of treatments should be carefully weighed against the possibility of close follow-up with treatments directed at controlling symptoms. Further investigation will be needed to capture the indolent and complex course these tumors typically experience.

## List of Abbreviations

*PGs*: Paragangliomas; *MDACC*: MD Anderson Cancer Center; *EBRT*: External beam radiation therapy; *CVAD*: Cyclophosphamide, vincristine, doxorubicin, and dacarbazine.

## Competing interests

The authors declare that they have no competing interests.

## Authors' contributions

DJM performed the review and collected the data, participated in the design of the study and drafted the manuscript. JRS assisted with reviewing and collecting the data for the study, took part in its design and helped draft the document. DS helped to collect and review the data, and also participated in the design. CJ offered assistance in drafting the manuscript. MDW also collected and reviewed the data and facilitated the draft. EYH conceived the study and aided in its design, as well as helping draft the document. MEK carried out the analysis, assisted in conception of the study and its design, and worked in drafting the manuscript. All authors read and approved the final document.
